# An Assessment of Systemic Factors and COVID-19 Mortality in Africa

**DOI:** 10.3389/ijph.2022.1604915

**Published:** 2022-09-13

**Authors:** Ayomide Owoyemi, Tolulope Balogun, Joy Okoro, Tariro Ndoro, Oluwakayode Fasominu, Adejare Atanda, Ibraheem Abioye

**Affiliations:** ^1^ Department of Biomedical and Health Information Sciences, University of Illinois at Chicago, Chicago, IL, United States; ^2^ Royal Surrey County Hospital, Guildford, United Kingdom; ^3^ Quillen College of Medicine, East Tennessee State University, Johnson City, TN, United States; ^4^ Department of Epidemiology and Biostatistics, University of the Witwatersrand, Johannesburg, South Africa; ^5^ UNICEF United Nations International Children’s Emergency Fund, New York, NY, United States; ^6^ Ending Pandemics, San Francisco, CA, United States; ^7^ Department of Global Health and Population, School of Public Health, Harvard University, Boston, MA, United States

**Keywords:** mortality, COVID, lockdown, governance, Africa, stringency index

## Abstract

**Objectives:** The objective of this study was to examine the association between several country-level systemic indices and the deaths from COVID-19 across African countries.

**Method:** Regression analyses were conducted to test the association between selected indices and deaths from COVID-19 across African countries. All tests were run at the *α* = 0.05 level of significance.

**Result:** We found a statistically significant correlation between total COVID-19 deaths per million and Stringency Index (*p*-value <0.001) and Human Development Index (*p*-value <0.001). Multiple regression analysis showed that Stringency Index was the only variable that remained significant when other factors are controlled for in the model.

**Conclusion:** Countries in Africa with poorer governance, inadequate pandemic preparedness and lower levels of development have unexpectedly fared better with respect to COVID-19 deaths mainly because of having a younger population than the countries with better indices.

## Introduction

The first case of the novel coronavirus disease (COVID-19) was reported in Wuhan, China. The disease is caused by the Severe Acute Respiratory Syndrome coronavirus 2 (SARS-CoV-2). On the African continent, the first case of the disease was confirmed in Egypt on the 14th of February 2020 [1]. Since then, the infection has spread to several African countries. As of February 2022, there have been more than 11.1 million total cases and over 247,000 mortalities recorded across Africa [2]. While vaccines are being rapidly rolled-out, non-pharmaceutical interventions like the closure of schools, travel restrictions, bans on public gatherings, and stay-at-home (lockdown) orders etc. are still being applied to varying degrees to manage the spread of the virus [3, 4]. These strategies are aimed at reducing the spread of the disease and protecting high-risk individuals and have varied across the continent in the degree of implementation and effectiveness based on differing country dynamics [5, 6].

African countries have some of the poorest health indices in the world. This is evident in the poor performance of their health systems and the ineffectiveness of their governance systems [7]. The continent also houses the largest percentage of the poorest people in the world and has had challenges in the progress towards achieving the Sustainable Development Goals [8]. These factors have an impact on the effectiveness of response efforts [9]. While Africa has not recorded high mortalities in comparison to Europe and South America, reported mortality has varied across the continent [10]. Cumulative confirmed deaths in Africa as of February 2022 was 239,838 for Africa and 1.63 Million and 1.22 million for Europe and America, respectively [11].

COVID-19 mortality across the globe has been shown to vary between countries. Non-pharmaceutical measures have been shown to be effective in curbing the transmission of the virus, and by extension reduce the mortalities associated with the pandemic, and the effect of this has varied by the level of stringency of these measures and the time of implementation [12, 13]. The mortality from COVID-19 has also varied due to other factors which include proportion of the elderly in the population, prevalence of co-morbidities like malignancies, diabetes, and social and systemic factors which include the level of health system preparedness, pandemic response time, the stringency of the non-pharmaceutical preventive measures, the effectiveness of governance [12, 14, 15].

These systemic factors especially the ones dealing with the quality of governance, pandemic preparedness, quality of healthcare, and level of human development have been shown to be highly relevant to the management of disease outbreaks and overall healthcare outcomes across the world [16–19]. For the convenience of nomenclature for this study these factors will all be collectively referred to as systemic factors.

Several indices have been developed to measure and assess some of these selected systemic factors, these include Government Response Stringency Index (SI), Human Development Index (HDI), Global Health Security Index (GHSI) and the Ibrahim Index of African Governance (IIAG). The Government Response Stringency Index (SI) was created by the Oxford research group as a composite measure of restriction policies made towards limiting the spread of the virus [20]. Two recent studies have revealed that lower degree of government stringency and slower response times were associated with more deaths from COVID-19 while the level of preparedness for epidemics had no effect on recorded deaths [21, 22].

The Global Health Security Index (GHSI) is a comprehensive assessment of capabilities in different countries to prevent and respond to the threats of infectious diseases, it was developed by the Nuclear Threat Initiative (NTI), Johns Hopkins Center for Health Security (JHU) and the Economist Intelligence Unit (EIU) [23]. A study of the GHSI of Organization for Economic Cooperation and Development OECD countries’ performance during the pandemic showed a discrepancy in GHSI rating of some countries and their actual performance as some countries with high GHSI scores did not fare well in managing the spread of COVID-19 [24].

The Human Development Index (HDI) was developed by the United Nations Development Program (UNDP) to measure the aspects of human development and standards of living in member states [25]. Studies conducted by Liu et al and Imtyaz et al demonstrated a positive relationship between high development index and higher death outcomes for COVID-19 [26, 27]. Considering the significance of governance and government capacity to appropriately responding to threats and matters, the Ibrahim Index of African Governance (IIAG) is a useful metric to consider in understanding how effective African governments are in in this regard [28]. The Mo Ibrahim foundation that is behind the index released a report on COVID-19 in Africa highlighting challenges with managing the pandemic on the continent which include poor access to healthcare, inadequate infrastructure, poor data practices and weak health systems [29].

There is a paucity of studies addressing the impact of these systemic factors on COVID death outcomes on the continent. This study aims to examine the association between systemic factors using indices like SI, GHSI, HDI, IIAG and the deaths from COVID-19 across African countries. Understanding how these selected systemic factors correlate with deaths due to COVID-19 can help inform decisions on system strengthening policies and governance across the continent in managing the pandemic and other infectious diseases that plague the continent. Understanding such associations can also help emphasize the importance of the state capacity, state response, and the quality of governance in effective epidemic response and management.

## Methods

### Study Design

We conducted an ecological analysis of COVID-19 mortality and selected governance variables across 54 African countries. This included data available data from when the pandemic began up to the 31st of October 2020. The end date was chosen based on when most African countries had lifted lockdown measures.

### Data Collection

Data were obtained from openly available sources. The COVID-19, population and Human Development Index data were obtained from; Our World in Data website which aggregates COVID-19 data from the European Centre for Disease Prevention and Control (ECDC) [10]. The dataset consisted of aggregated data from various sources such as World Health Organization (WHO), European Center for Disease Prevention and Control, the Centers for Disease Control and Prevention (CDC), and various other governmental and non-governmental organizations. The data covers 54 countries on the African continent. The Health Security Index data were obtained from the Global Health Security Index database, while the Ibrahim Index of African Governance (IIAG) index data were obtained from the IIAG website [23, 28].

### Data Analysis

We applied linear and multiple regression to analyze the association between the dependent variable which is deaths from COVID-19 (total COVID-19 deaths per million), and the independent variables which are Government Response Stringency Index, Global Health Security Index, Human Development Index, and Ibrahim Index of African Governance, with percentage of population aged above 65 as the covariate. The total deaths per millions was used for this analysis to ensure an adjustment for population density differences for the COVID-19 deaths across the different countries. For Stringency Index 18 countries were excluded as there was no data available for them.

We log-transformed the total COVID-19 deaths per million (TCDPerMill) outcome variable, using log-linear regression to examine the relationship between the outcome and independent variables and to ensure that we passed the assumptions of simple and multiple linear regression. This was done to normalize the data and manage the skewness of the data. All tests were run at the *α* = 0.05 level of significance. We also conducted an analysis of variance to evaluate and validate the model. To determine the overall regression equation, we used the ***Unstandardized coefficients*** to see the effects of each of the independent variables on the outcome. All statistical analyses were conducted using SAS software 9.4 (SAS Institute Inc., Cary NC).

## Results

Data on TCDpermill, percentage of population aged 65 and older, SI, GHSI, IIAG and HDI data for 54 African countries are shown in Table 1.

**TABLE 1:** TCDpermill, percentage of population aged 65 and older, SI, GHSI, IIAG and HDI data for African countries (An Assessment of Systemic Factors and COVID-19 Mortality in Africa, Africa, 2021).

Country	TCDperMill	Age65older	SI	GHSI	IIAG	HDI
Algeria	44.606	6.211	75	23.6	56.2	0.754
Angola	8.367	2.405	65.74	25.2	40	0.581
Benin	3.382	3.244	43.52	28.8	58.6	0.515
Botswana	10.206	3.941	52.78	31.1	66.9	0.717
Burkina Faso	3.205	2.409	22.22	30.1	54	0.423
Burundi	0.084	2.562	14.81	22.8	36.9	0.417
Cameroon	16.048	3.165	45.37	34.4	43.5	0.556
Cape Verde	170.867	4.46	71.3	29.3	73.1	0.654
Central African Republic	12.837	3.655		27.3	30.7	0.367
Chad	5.966	2.486	66.67	28.8	33.9	0.404
Comoros	8.05	2.963		27.2	43.2	0.503
Congo	16.672	3.402	47.22	23.6	36.1	0.606
Cote d'Ivoire	4.701	2.933		35.5	53.9	0.492
Democratic Republic of Congo	3.417	3.02		26.5	31.7	0.457
Djibouti	61.741	4.213	43.52	23.2	41.3	0.476
Egypt	61.152	5.159	75.93	39.9	47.4	0.696
Equatorial Guinea	59.16	2.846		16.2	28.7	0.591
Eritrea		3.607	81.48	22.4	25.8	0.44
Ethiopia	12.734	3.526	57.41	40.6	46.6	0.463
Gabon	24.711	4.45	69.44	20	47.7	0.702
Gambia	49.241	2.339	56.48	34.2	55.9	0.46
Ghana	10.298	3.385	38.89	35.5	64.3	0.592
Guinea	5.482	3.135	35.65	32.7	42.5	0.459
Guinea-Bissau	20.833	3.002		20	41.4	0.455
Kenya	18.244	2.686	68.52	47.1	58.5	0.59
Lesotho	20.072	4.506	40.74	30.2	52.3	0.52
Liberia	16.213	3.057	57.41	35.1	47.9	0.435
Libya	123.267	4.424	79.63	25.7	35.2	0.706
Madagascar	8.812	2.929	52.78	40.1	44.4	0.519
Malawi	9.618	2.979	50.93	28	51.5	0.477
Mali	6.716	2.519	34.26	29	46.6	0.427
Mauritania	35.056	3.138		27.5	41.6	0.52
Mauritius	7.863	10.945		34.9	77.2	0.79
Morocco	98.21	6.769	65.74	43.7	61	0.667
Mozambique	2.911	3.158	53.7	28.1	49	0.437
Namibia	52.343	3.552		35.6	65.1	0.647
Niger	2.85	2.553	11.11	32.2	47.8	0.354
Nigeria	5.55	2.751	50.93	37.8	45.5	0.532
Rwanda	2.702	2.974		34.2	60.5	0.524
Sao Tome and Principe	73.006	2.886		17.7	60.4	0.589
Senegal	19.291	3.008	37.96	37.9	63.2	0.505
Seychelles		8.606		31.9	72.3	0.797
Sierra Leone	9.277	2.538	31.48	38.2	51	0.419
Somalia	6.544	2.731	32.41	16.6	19.2	
South Africa	324.236	5.344		54.8	65.8	0.699
South Sudan	5.181	3.441	35.19	21.7	20.7	0.388
Sudan	19.088	3.548	32.41	26.2	32.5	0.502
eSwatini (Swaziland)	100.848	3.163		31.1	43.8	0.588
Tanzania	0.352	3.108		36.4	53	0.538
Togo	6.644	2.839		32.5	50.1	0.503
Tunisia	111.434	8.001	75	33.7	70.4	0.735
Uganda	2.405	2.168	57.41	44.3	51.8	0.516
Zambia	18.984	2.48		28.7	52	0.588
Zimbabwe	16.282	2.822		38.2	46.1	0.535

The regression equation predicting TCDPerMill from all the independent variables is as follows:

TCDPerMill=−0.637+0.188×Age56Older+0.016×SI+0.002×GHSI+0.005×IIAG−0.0126×HDI

**FIGURE 1:**
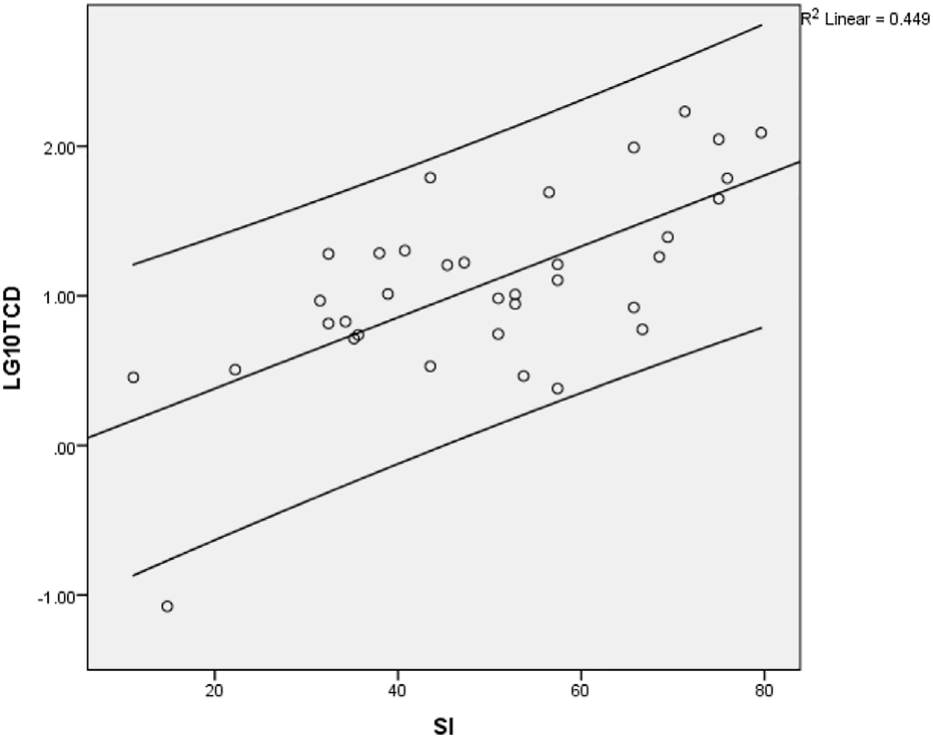
Relationship between stringency index and Total Covid Deaths per million. The dependent variable used here is the log_10_ of TCDPerMill (An Assessment of Systemic Factors and COVID-19 Mortality in Africa, Africa, 2021).

The Regression analysis (Figure 1) showed a statistically significant correlation between TCDPerMill and SI (coefficient = 0.024, *p*-value −0.000), and HDI relationship (coefficient = 3.303, *p*-value 0.000). No statistically significant relationship (coefficient = 0.007, *p*-value 0.537) was also found between TCDPerMill and GHSI (coefficient = 0.007, *p*-value 0.537), and IIAG (coefficient = 0.012, *p*-value 0.094).

Multiple linear regression was carried out to investigate whether SI, GHSI, HDI, IIAG and percentage of population aged above 65 could predict COVID-19 deaths in African countries. The results (Table 2) of the regression showed that the model was a significant predictor of COVID-19 deaths (F [5, 28] = 7.586, *p* = 0.00001). SI was the only variables that was significant in the model. Based on the model SI yields a 0.46 increase in TCDperMillion for every unit increase while controlling for the other variables and the covariate (Age65Older).

**TABLE 2:** Results of multiple linear regression of the independent variables against Total Covid Deaths per million (An Assessment of Systemic Factors and COVID-19 Mortality in Africa, Africa, 2021).

(Constant)	−0.637 (0.536)
Age65older	0.188 (0.086)
SI	0.016^a^ (0.007)
GHSI	0.002 (0.013)
IIAG	0.005 (0.009)
HDI	−0.126 (1.321)
R- Squared	0.575
No of Observations	54

Standard errors are in parentheses.

The dependent variable used here is the log_10_ of TCDPerMill, and Age65older is the covariate.

a Indicates statistical significance.

## Discussion

This study provides insight into factors affecting recorded deaths attributed COVID-19 across Africa and helps to further our understanding of how governance and health indices are associated with the recorded mortalities from the COVID-19 pandemic. Statistically significant associations were not found between the GHSI, IIAG, and deaths from COVID-19 which is similar to results obtained from a study conducted by Hooper that showed that the GHSI had no effects on COVID-19 deaths [21]. However, there was a positive association between deaths from COVID-19, SI and HDI. The GHSI is known as a tool that shows promise in giving insights into levels of biosafety and levels of systems preparedness for countries, but they are encouraged to look beyond it for mitigating factors for impact of pandemics, as this study and another conducted by Hooper have shown how it has no effect on the death outcomes for COVID-19 [21, 30]. The GHSI has been criticized for not incorporating the social and political features that have significant effect on public health outcomes across different countries, hence this is why it has not performed well in aligning with performance of different countries in managing COVID-19 [31].

Some of these results can be explained by the fact that we used total deaths as opposed to weekly averages for this study and the possibility that countries that had higher mortalities implemented stricter or more stringent measures to control the pandemic. The positive association seen between HDI and COVID-19 deaths is underlined by the percentage of the population aged above 65 being higher in countries with higher levels of HDI, because these countries have higher life expectancy because of better healthcare and living conditions [32]. Also, since these countries have higher indices of SI and IIAG too, and COVID-19 is more fatal in individuals above the age of 65, the positive association can be explained by the fact that there will be more deaths in these countries because of their higher population of persons above 65, and older people also constitute a significant percentage of those with co-morbidities [33, 34]. This is consistent with results from a study conducted by Liu et al which revealed a positive association between COVID-19 deaths and HDI in Italy, and in another study by Imtyaz et al. that revealed that European countries were recording higher COVID-19 deaths due to higher percentage of elderly people compared to other countries [26, 27].

However, the impact of HDI disappeared when we controlled for variables in a multiple regression with just Stringency Index and percentage of population aged 65 and above showing significance. This disappearance of effect could be explained by the fact that countries with higher HDIs have larger percentages of the population above 65 and controlling for this variable removes that effect. As shown earlier that there is a linear relationship between HDI and COVID-19 deaths, this effect can then be explained by the percentage of population aged above 65 in each country. This also shows that the strongest factors related to outcomes of death in Africa are SI and percentage of the population aged above 65.

### Limitations

Our findings should be considered in the context of several limitations. Our study is subject to inherent ecological fallacies that stem from the data used which are country-level data such as total deaths which is highly affected by the variability in COVID-19 reporting, between countries in Africa. Also, a more appropriate way to ascertain the effect of SI will be to assess its relationships with weekly moving averages over time as this will show how recorded deaths have changed and if stringency measures have been effective. We were furthermore unable to account for the time to implementation of the social distancing measures, and death outcomes which will differ based on how soon each country implemented their measures. It is also important to note that our results are only showing associations and not inferring causality. This is important context for interpreting and applying the lessons from this study.

### Conclusion

COVID-19 deaths on the African continent have largely been less compared to other countries and places in the world. Countries in Africa with poorer governance, inadequate pandemic preparedness and lower levels of development have unexpectedly fared better with respect to COVID-19 deaths mainly because of having a younger population than the countries with better indices. This study helps with insight on one of the most significant reasons that is associated with higher COVID-19 deaths per population across African countries which is population demographics.
